# Effect of the Fabrication Parameters of the Nanosphere Lithography Method on the Properties of the Deposited Au-Ag Nanoparticle Arrays

**DOI:** 10.3390/ma10040381

**Published:** 2017-04-03

**Authors:** Jing Liu, Chaoyang Chen, Guangsong Yang, Yushan Chen, Cheng-Fu Yang

**Affiliations:** 1 School of Information Engineering, Jimei University, Xiamen 361021, China; jingliu@jmu.edu.cn (J.L.); xx002@jmu.edu.cn (C.C.); gsyang@jmu.edu.cn (G.Y.); chenys@jmu.edu.cn (Y.C.); 2 Department of Chemical and Materials Engineering, National University of Kaohsiung, No. 700, Kaohsiung University Rd., Nan-Tzu District, Kaohsiung 811, Taiwan

**Keywords:** fabrication parameter, Au-Ag nanoparticle arrays, nanosphere lithography (NSL)

## Abstract

The nanosphere lithography (NSL) method can be developed to deposit the Au-Ag triangle hexagonal nanoparticle arrays for the generation of localized surface plasmon resonance. Previously, we have found that the parameters used to form the NSL masks and the physical methods required to deposit the Au-Ag thin films had large effects on the geometry properties of the nanoparticle arrays. Considering this, the different parameters used to grow the Au-Ag triangle hexagonal nanoparticle arrays were investigated. A single-layer NSL mask was formed by using self-assembly nano-scale polystyrene (PS) nanospheres with an average radius of 265 nm. At first, the concentration of the nano-scale PS nanospheres in the solution was set at 6 wt %. Two coating methods, drop-coating and spin-coating, were used to coat the nano-scale PS nanospheres as a single-layer NSL mask. From the observations of scanning electronic microscopy (SEM), we found that the matrixes of the PS nanosphere masks fabricated by using the drop-coating method were more uniform and exhibited a smaller gap than those fabricated by the spin-coating method. Next, the drop-coating method was used to form the single-layer NSL mask and the concentration of nano-scale PS nanospheres in a solution that was changed from 4 to 10 wt %, for further study. The SEM images showed that when the concentrations of PS nanospheres in the solution were 6 and 8 wt %, the matrixes of the PS nanosphere masks were more uniform than those of 4 and 10 wt %. The effects of the one-side lifting angle of substrates and the vaporization temperature for the solvent of one-layer self-assembly PS nanosphere thin films, were also investigated. Finally, the concentration of the nano-scale PS nanospheres in the solution was set at 8 wt % to form the PS nanosphere masks by the drop-coating method. Three different physical deposition methods, including thermal evaporation, radio-frequency magnetron sputtering, and e-gun deposition, were used to deposit the Au-Ag triangle hexagonal periodic nanoparticle arrays. The SEM images showed that as the single-layer PS nanosphere mask was well controlled, the thermal evaporation could deposit the Au-Ag triangle hexagonal nanoparticle arrays with a higher quality than the other two methods.

## 1. Introduction

Surface plasmons are charge-density waves that will propagate over the surfaces of metals and reflect from discontinuities in the electric permittivity of either the metal or the overlying dielectric. If a metal thin film has two parallel boundaries, the surface plasmon waves will reflect at particular frequencies. The surface plasmons will form standing waves on the metal nanoparticles with sizes smaller than the wavelength of light, which are known as localized surface plasmon resonances (LSPRs). LSPRs are the optical phenomena generated by light when they interact with conductive metals or nanoparticles (NPs) that are smaller than the incident wavelengths. LSPRs are non-propagating excitations of the conduction electrons of metallic nanoparticles coupled to the electromagnetic field [1]. Since the first application of the surface plasmon resonance (SPR) phenomenon for gas detection and a biological sensor was in 1982 [2], the SPR sensing technology has been widely used for the detection of biological and chemical analytes, environmental monitoring, and medical diagnostics [3,4,5,6,7] in the past two decades. The underlying phenomena exploited for nanobiophotonic sensing applications is based on the interaction of light and matters at nanoscale surfaces.

As we know, many different structures are investigated to obtain the characteristics of the LSPRs in a matrix. For example, Brolo and co-workers were the first group to use arrays of sub-wavelength nanoholes in a gold thin film to monitor the binding of organic and biological molecules on a metallic surface [8]. This technique is based on the enhanced transmission of surface plasmon resonances through nanoholes in a collinear optical arrangement. Any change in the surface composition or geometry of the nanoholes is accompanied by a shift in the wavelength of the surface plasmon polarizations. Moreover, Steele and Brett used the Ag spheres and tilt nanowires, Si chevrons, and helical posts, to design LSPR devices [9], and Huang et al. investigated nanotube matrixes for LSPR devices [10]. Yamamichi et al used colloidal gold as nanometric metal particles to study the influence of the design structure and interparticle spacing on the sensing abilities [11].

Noble metal nanoparticles exhibit rich LSPR properties. Typical metals that support surface plasmon resonances are silver (Ag) [12], gold (Au) [13], and copper (Cu) [14] single-layer thin films, and hybrid Ni/Au [15] bi-layer thin films. For fabricating the Ag nanoparticle arrays, a nanoparticle monomer with different shapes has been shown to display a single dipole resonance peak, for example, a sphere, a cube, a tetrahedron, an octahedron, a triangular plate, and rectangular bars with different aspect ratios, respectively. By analyzing the spectral properties of silver nanoparticles in solution, it becomes clear that, when the diameters are greater than 80 nm, a second peak becomes visible at a shorter wavelength than the primary peak. This secondary peak is due to a quadrupole resonance that has a different electron oscillation pattern than the primary dipole resonance. However, most LSPR devices are designed on the basis of silver (Ag) thin-film metallic nanostructures. The corrosion originates from oxidation and sulfuration, which is well known for metal Ag thin films. Corrosion-induced electrochemical damage on the surface of the Ag thin films exists in an ambient atmosphere, especially over a period of time. However, the relative permittivity of the Ag thin films will be changed due to oxidation and sulfuration [16], and the optical properties of the nanophotonic devices will be changed. To overcome this problem, we put forth a hybrid Au-Ag nanostructure array with Au thin films which cover the surfaces of the Ag thin films [16,17]. Additionally, Cr thin films can be used as the interlayer [12,17] to improve the effect of nanoparticle arrays. In the past, the electron-beam lithography (EBL) [18] and photolithography [19] methods have been developed to fabricate the nanoparticle arrays for LSPR structures. 

The nanosphere lithography (NSL) method is a powerful, low-cost, and high-efficiency fabrication technique for the creation of nanoparticles or nanoparticle arrays with a controlled size, shape, and interparticle spacing. The NSL method can be sued to fabricate nanoparticle arrays with periodically and geometrically tunable structures [17,20,21,22]. For example, Zhu et al. used the NSL method to fabricate the hybrid Au-Ag triangular nanoparticles array [23], Ag hemisphere nanoparticle array [24], and Ag rhombic nanoparticles [25]. For the NSL method, the nano-scale PS nanospheres can be used as masks and the physical deposition methods can be used to deposit the metal thin films with the nanoparticle arrays. Also, as the NSL method was used, only a few papers have reported the effects of the fabrication parameters of self-assembly nano-scale polystyrene (PS) nanospheres on the properties of the single-layer or hybrid multi-layer metal nanoparticle arrays [12,17]. In this study, we first compared the drop-coating and spin-coating methods, and used the solution with 6 wt % PS nanospheres to construct the single-layer NSL masks by using the self-assembly method of nano-scale PS nanospheres. We found that the metal thin films deposited using the masks formed by the drop-coating method constructed the Au-Ag thin films with better geometric properties. Next, the drop-coating method was used to deposit the PS nanospheres for constructing the NSL masks, and the concentration of the nano-scale PS nanospheres in solution was changed from 4 to 10 wt % and the angle of the one-side lifting table was changed from 0° to 15°. Thermal evaporation was used to deposit the Au-Ag triangle hexagonal nanoparticle arrays in the two deposition parameters. Scanning electronic microscopy (SEM) was used to observe the surface morphologies of the PS nanosphere masks formed by the self-assembly method and the deposited Au-Ag triangle hexagonal nanoparticle arrays. We compared the uniformities of the Au-Ag triangle hexagonal nanoparticle arrays by using the SEM images of surface observations. Finally, we also compared the properties of the Au-Ag triangle hexagonal nanoparticle arrays by using the differently physical deposition methods of thermal evaporation, radio-frequency (RF) magnetron sputtering, and e-gun deposition, respectively. 

## 2. Fabrication of Nanostructures

**Figure 1:**
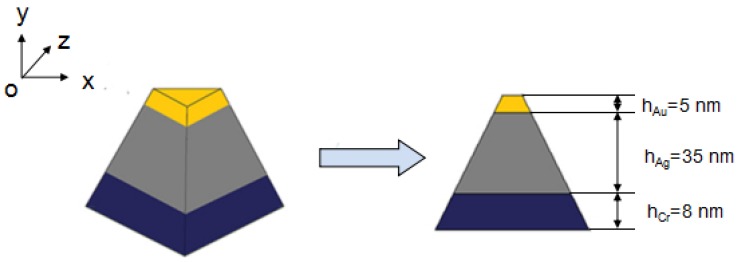
A monomer model of the triangle Ag nanoparticle arrays in 3D view and side cross-section view.

In the past, we have obtained a suitable thickness of Cr interlayer thin films according to theoretical calculations and experimental results [17]. Figure 1 illustrates the schematic side view for the design structure of the Au-Ag nanoparticle arrays. The thicknesses of Cr, Ag, and Au thin films are defined in Figure 1, and silicon was used as the substrate. A corresponding schematic illustration of the Au-Ag nanoprism, the monomer model of the Au-Ag nanoparticle arrays, was the equilateral triangle. The out-of-plane heights of the Cr, Ag, and Au nanoparticles under the Au layer were 8, 35, and 5 nm, respectively.

**Figure 2:**
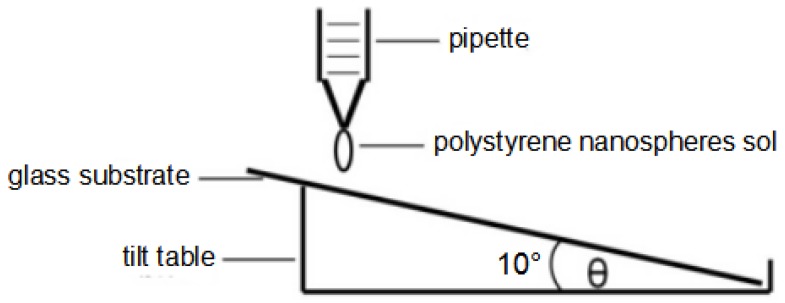
Schematic diagram illustrating the drop-coating method completing the self-assembly arrangement.

At first, the PS nanospheres with a mean radius of 265 nm and a concentration of 10 wt % in solution, were purchased from Suzhou Nano-Micro Bio-Tech Co. Ltd. (Suzhou, China). As we know, the construction of close packed nanospheres was a prerequisite for forming the NSL masks. The silicon substrates (n-type, (100) orientation) were ultrasonic cleaned in toluene, acetone, and ethanol for 10 min, and then in piranha solution (H_2_SO_4_:H_2_O_2_ = 3:1) for 2 h, to remove organic residues. The details used to prepare the substrates are described in [17]. The regular monolayer PS nanospheres as a deposition mask were critical for achieving a large-area hexagonal structure. To begin with, the PS nanosphere solution was diluted to 6 wt % with deionized water. The drop-coating and spin-coating methods were the first variable factors used to prepare the deposition masks. The specific steps were as follows:

(1) We used the drop-coating method to complete the fabrication of the deposition masks on the first group of substrates, before placing the substrates on a one-side lifting table with an angle of 10°, as shown in Figure 2. We used a pipette to draw the solution of PS nanospheres on the substrates and fixed the angle for 20 min; the solution of PS nanospheres drawn for each sample was about 10 μL. Following this, the substrates were placed in an electric thermostat blast drying oven for 30 min. The temperature in the oven was kept at 50 °C for evaporation drying and the angle was maintained during the heating process.
(2) We used the spin-coating method to complete the fabrication of the deposition masks on the second group of substrates. A KW-4A high speed spin coater was used as the coating equipment, which was fabricated by Xiamen McLaren fine Ruike Instrument Co., Ltd. (Xiamen, China). The solution of PS nanospheres was dropped onto the substrates, and the substrates were then placed on the spin coater. To begin with, the high speed spin machine was carried out for 5 s at a low spin speed (500 rpm) and the next was carried out for 20 s at a high spin speed (2000 rpm). The parameters of the spin coater were the optimum values identified after we had carried out many experimental processes. Additionally, the substrates were placed in an electric thermostat blast drying oven and the temperature in the oven was kept at 50 °C for evaporation drying.

The depositions of Au (99.9%), Ag (99.9%), and Cr (99.9%) were performed in a home-built thermal evaporator at a pressure of 5.0 × 10^−4^ Pa. The granulated metallic source materials were placed in molybdenum boats (1 cm^2^) that directly faced toward the substrate, at a distance of 16.0 cm. During the deposition process, the substrates were rotated at a frequency of 16.5 rpm. After the PS nanosphere masks were formed by using the drop-coating method and spin-coating method, Cr, Ag, and Au thin films were deposited on the PS nanosphere-coated substrates, to construct the Au-Ag triangle hexagonal nanoparticle arrays by the thermal evaporator. The power for used for the heating-up of the source materials was carefully increased, in order to achieve a homogeneous deposition. The deposition of the Cr, Ag, and Au metallic layers was performed at a pressure of 5.0 × 10^−4^ Pa, and the deposition rate was 4.0 nm/s for Cr thin films and 2.5 nm/s for Ag and Au thin films. For comparative purposes, the drop-coating method was used to prepare the deposition masks, and the concentration of the nano-scale PS nanospheres in the solution and the one-side lifting angle were used to identify the optimum parameters for constructing NSL masks. Thermal evaporation, RF magnetron sputtering, and e-gun deposition were used to deposit the Au-Ag triangle hexagonal nanoparticle arrays for finding the optimal physical method for growing the hybrid Au-Ag thin films. The deposition parameters for the thermal evaporator have previously been mentioned. For RF magnetron sputtering and e-gun deposition, the deposition of metallic layers was performed at a pressure of 5.0 × 10^−4^ Pa, and the deposition rates of the Ag and Au thin films were 0.11 and 0.27 nm/s, respectively. The thicknesses of Cr (h_cr_), Ag (h_Ag_), and Au (h_A__u_), of 8, 35, and 5 nm respectively, were produced by controlling the deposition time. The PS nanosphere masks were removed by sonication (B3500S-MT, 140 W, 42 kHz, Branson Ultrasonics, Danbury, CT, USA) in absolute ethanol, to examine the adhesive ability of the Au-Ag-Cr nanoparticles on the substrates. After Cr, Ag, and Au thin films were deposited on the substrates, there were soaked in a beaker with absolute ethanol solution. The beaker was placed in the sonication tank and was ultrasonically shocked in high power mode for 5 s. The achieved PS masks and the structures of the constructed Au-Ag-Cr nanoparticle arrays were characterized by scanning electron microscopy (SEM, LEO-1530, Zeiss, Oberkochen, Germany).

## 3. Results and Discussion

### 3.1. The Selection of the Drop-Coating Method and Spin-Coating Method

As the NSL method was used to fabricate the hybrid Au-Ag nanoparticle arrays, drop-coating [26] and spin-coating [27] were the two methods used to fabricate the PS masks. Figure 2 is the schematic diagram for the drop-coating method. The accomplishment of the self-assembly process by using the drop-coating method was mainly controlled by two factors. First, the effect of gravity existed, because there is a certain inclination angle between the substrate and horizontal plane. Furthermore, when the solution is evaporated at a certain temperature, particle surface tension arises. The use of a spin-coating method to complete the fabrication of PS nanosphere masks is mainly controlled by inertia force provided by the coater, which is similar to the gravity and surface tension in the drop-coating method. The spin-coating method was also used as the self-assembly method to fabricate the masks. First, the solution of PS nanospheres was dropped onto the substrates, and the substrates were then lightly placed on the coater. The high-speed rotation of the coater caused the PS nanospheres to move to the edge of the substrates, and next, the PS nanospheres were slowly arranged from the edge to the central position. Under the capillary force between the particles, the PS nanospheres were accumulated into a hexagonal close-packed structure with the lowest amount of free energy.

**Figure 3:**
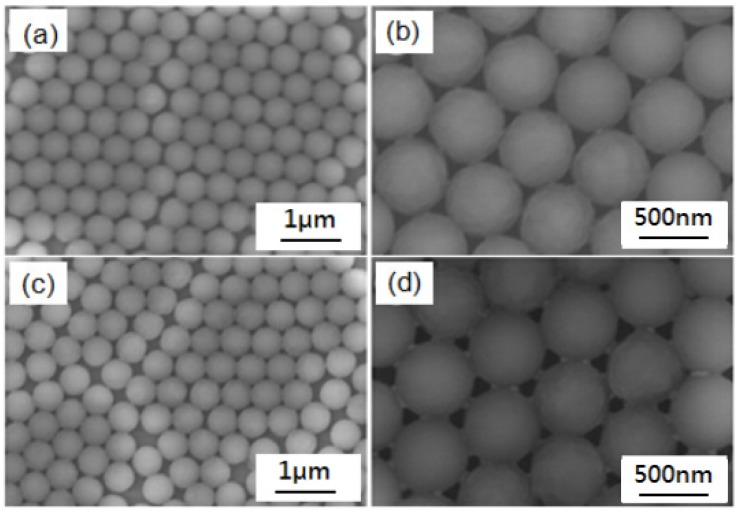

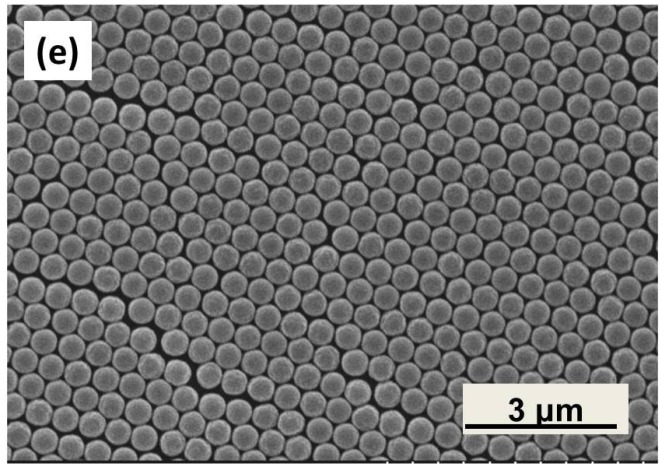
SEM images of the self-assembly PS nanosphere masks fabricated by different coating methods. (**a**,**b**) drop-coating self-assembly method; (**c**,**d**) spin-coating self-assembly method; (**e**) drop-coating self-assembly method in a large area.

Therefore, under the same circumstances, we used the drop-coating and spin-coating method to complete the self-assembly experiments of PS nanosphere masks, and to observe the corresponding deposition masks. Figure 3 shows the SEM images of the self-assembly PS nanosphere masks fabricated by the drop-coating (with one-side lifting angle of 10°) and spin-coating methods, for which the vaporization temperature of the prepared samples was 55 °C. Figure 3a,b are different magnification SEM images of masks fabricated by the drop-coating method and Figure 3c,d are different magnification SEM images of the PS nanosphere masks fabricated by the spin-coating method. For a small area of the PS nanosphere masks, as Figure 3b,d show, the experimental results show that both the drop-coating and spin-coating method could be used to fabricate nanosphere masks, which presented hexagonal close-packed structures and met the ideal construction required by the NSL method. However, for the PS nanosphere masks in a larger area, although the PS nanosphere masks fabricated by the spin-coating method could present a single-layer regular arrangement, there were still many defects in the mask, as Figure 3c shows. In contrast, the mask fabricated by the drop-coating method had a completely single-layer close-packed structure, as Figure 3a shows. 

**Figure 4:**
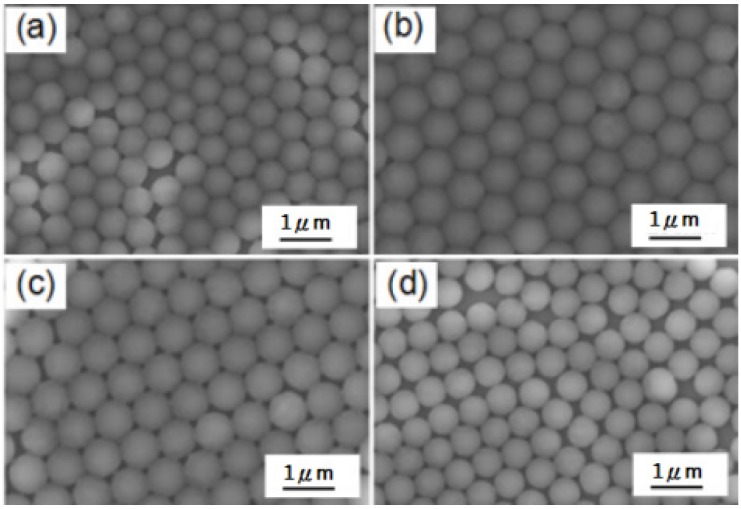
SEM images of self-assembly PS nanosphere masks fabricated with different one-side lifting angles of substrates. The angles used were (**a**) 0°; (**b**) 5°; (**c**) 10°; and (**d**) 15°.

This difference may be due to the spin-coating method, during which the suspension with the substrate occurs at a high rotation speed on the coater and the solution is rapidly rotating throughout. Although the inertial force can encourage the particles to approach each other, in some areas, the half crescent water layer is not formed. Thus, capillary forces between the particles cannot be formed and a hexagonal close-packed structure with the lowest amount of free energy cannot be obtained. As Figure 3e shows, the SEM image of the drop-coating self-assembly method in a large area proves that the hexagonal close-packed structure has been formed. For the spin-coating self-assembly method, the close-packed structure cannot be observed and only a structure similar to Figure 4c is observed (not shown here). However, we also believe that the arrangement of the PS nanosphere masks fabricated by the spin-coating method is not as regular as that fabricated by the drop-coating method. However, for experimental fabrication, the requirement of arrangement regularity is not too high and the spin-coating method can be adopted to fabricate the nanosphere masks. The advantage of the spin-coating method is that it can be used to fabricate nanosphere masks with a larger area, and this structure can then be used to form the large-area two-dimensional ordered noble metal nanoparticle arrays, which can build the foundation for the application of the NSL devices.

**Figure 5:**
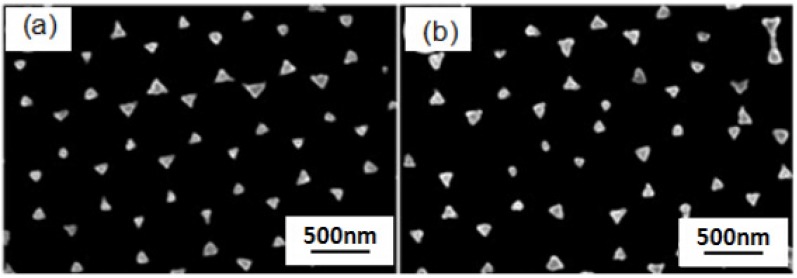
SEM images of two-dimensional ordered Au-Ag nanoparticle arrays as a function of the coating method of the PS nanosphere mask. (**a**) Drop-coating method; and (**b**) spin-coating method.

The Cr, Ag, and Au thin films are deposited on the two group samples shown in Figure 3 by thermal evaporation. After the PS nanosphere masks were removed by sonication, the two-dimensional Au-Ag nanoparticle arrays were obtained, as shown in Figure 5. It can be seen from Figure 5 that the whole structure can present periodic triangle hexagonal nanoparticle arrays. However, when the results in Figure 5 are compared, the periodic triangle hexagonal nanoparticle arrays fabricated by using the drop-coating mask are more complete, orderly, and are arranged in a more regular form than those fabricated by using the spin-coating mask. The distributions of the length of the sides of the Au-Ag triangle nanoparticles by using the drop-coating method and spin-coating method as masks were in the ranges of 50–80 and 35–95 nm, respectively.

From the perspective of the mechanism principle, in the experiments using the drop-coating method and spin-coating method, the obtained supporting forces of the PS nanospheres are different, which may lead to the different results in the structures of nanosphere masks, thus leading to the different results in the Au-Ag nanoparticle arrays. The results in Figure 5 show that Au-Ag thin films using nanosphere masks fabricated by the drop-coating method reveal more uniform nanoparticle arrays than those using masks fabricated by the spin-coating method. We used the drop-coating method to fabricate nano-scale PS nanosphere masks and to systematically investigate the fabrication parameters on the properties of the self-assembly nano-scale PS nanosphere masks, including the one-side lifting angle of the substrates and the vaporization temperature for the solvent of the self-assembly PS nanosphere thin films. Values of 0°, 5°, 10°, and 15° were used as one-side lifting angles, as Figure 4 shows, and we found that 10° was the optimum angle. When 0° was used as a one-side lifting angle, the PS nanospheres did not exhibit a large enough enough gravity force, so the self-assembly process was slow, and dislocation and a vacant space (or gap) were observed. The dislocation and vacant space decreased as the one-side lifting angle increased from 0° to 10°, and the self-assembly nano-scale PS nanosphere masks with a one-side lifting angle of 10° demonstrated the optimum uniformity and densification values, as Figure 4a–c show. As the one-side lifting angle was increased to 15°, the PS nanosphere masks had more vacancies and most PS nanospheres were not in orderly positions, as shown in Figure 4d. 

**Figure 6:**
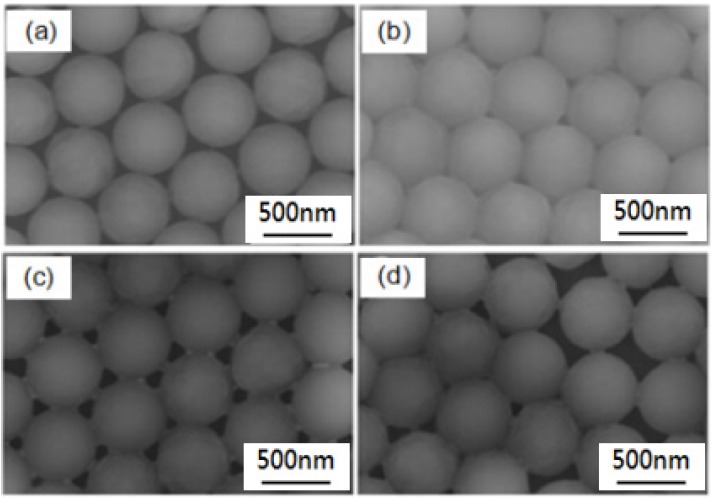
SEM images of self-assembly PS nanosphere masks fabricated with different evaporation temperatures during the self-assembly process. The evaporation temperature was (**a**) 25 °C; (**b**) 40 °C; (**c**) 55 °C; and (**d**) 70 °C.

The vaporization temperature for the solvent of the self-assembly PS nanosphere thin films was changed to 25, 40, 55, and 70 °C, respectively. The convection effect and capillary pressure are the most important factors for the self-assembly process. When a lower vaporization temperature (25 °C) was used, as Figure 6a shows, the solvent vaporized at a slower rate, and also had a slower moving velocity and a smaller convection effect. This means that the convection effect will vanish before the effect of capillary pressure to push nano-scale PS nanospheres moving for self-assembly, and a vacant space will appear in the matrix of PS nanosphere masks. When a higher vaporization temperature (70 °C) was used, as Figure 6d shows, the PS nanospheres had a higher free speed, and the solvent was vaporized before the capillary pressure to push nano-scale PS nanospheres moving for self-assembly, thus creating dislocations. We found that 40 and 55 °C were the better temperatures for the vaporization of solvent in the nano-scale the solution of PS nanospheres, as Figure 6b,c show. Considering this, used the drop-coating method with a one-side lifting angle of 10° and a vaporization temperature of 55 °C to construct the PS nanosphere masks for the following experiments.

### 3.2. Concentration of Nano-Scale PS Nanospheres in the Solution

**Figure 7:**
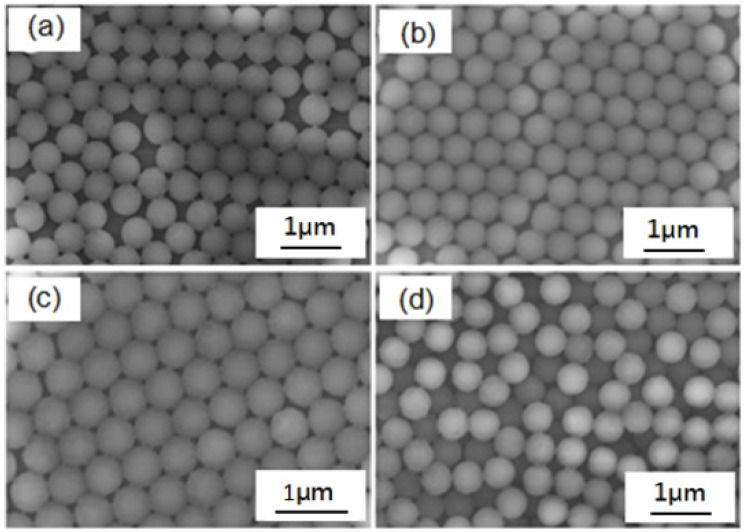
SEM images of self-assembly PS nanosphere masks fabricated as a function of the concentration of PS nanospheres in the dipping solution. (**a**) 4 wt %; (**b**) 6 wt %; (**c**) 8 wt %; and (**d**) 10 wt %, respectively.

When the drop-coating method is used to produce the self-assembly mask, the concentration is an important parameter which affects the order in which the PS nanosphere masks are arranged. For comparison, the concentration of PS nanospheres was changed to 4 wt %, 6 wt %, 8 wt %, and 10 wt %. When the concentration was too low, as Figure 7a shows for the concentration of 4 wt %, there were not enough PS nanospheres moving to the ordered location before the solvent was vaporized, resulting in the emergence of many dislocations and vacancies in the PS nanosphere masks. When the concentration was too high, as Figure 7d shows for the concentration of 10 wt %, the convection effect was limited and some PS nanospheres would stack in a small or local region to form the multi-layer PS nanosphere masks. The two results have apparent defects in the structure of the PS nanosphere masks, which will affect the properties of the deposited nanoparticle arrays. As the concentration was increased to 6 wt %, as Figure 7b shows, the PS nanosphere masks revealed a hexagonal close-packed structure and the dislocations and vacancies decreased. As the concentration was increased to 8 wt %, as Figure 7c shows, the PS nanosphere masks revealed a hexagonal close-packed and almost no dislocations and vacancies were observed. 

**Figure 8:**
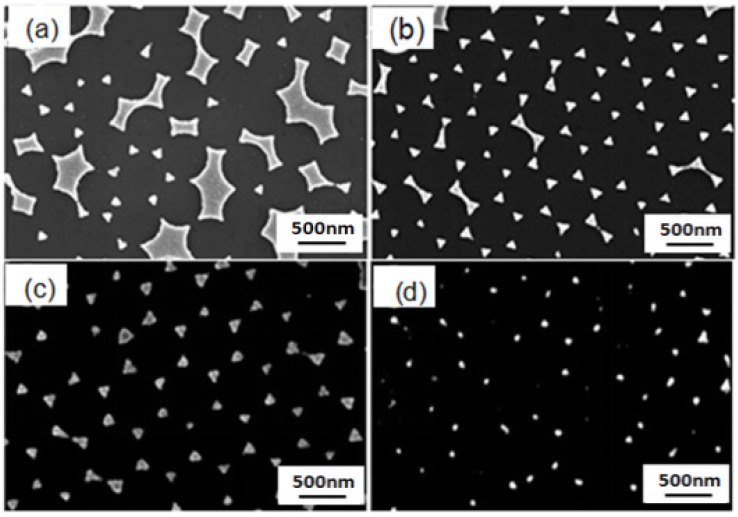
SEM images of two-dimensional ordered Au-Ag nanopariticle arrays as a function of concentration of PS nanospheres in the dipping solution. (**a**) 4 wt %; (**b**) 6 wt %; (**c**) 8 wt %; and (**d**) 10 wt %, respectively.

The self-assembly nanosphere masks shown in Figure 7 were used, and the Cr, Ag, and Au thin films were deposited on these masks by thermal evaporation. After the PS nanosphere masks were removed by sonication, two-dimensional Au-Ag triangle hexagonal nanoparticle arrays were obtained, as shown in Figure 8. The results in Figure 8 prove that the concentration (or called the masks’ properties) of PS nanospheres in the dipping solution is another important factor affecting the properties of the ordered Au-Ag nanopariticle arrays. As the masks were fabricated by using the solution with a concentration of 4 wt % PS nanospheres, as Figure 8a shows, the Au-Ag nanopariticle arrays were not in a regular format and many defects were observed. As the masks were fabricated by using the concentrations of 6 wt % and 8 wt % PS nanospheres, as Figure 8b,c show, the Au-Ag nanopariticle arrays were in a regular format and the defects in the Au-Ag nanopariticle arrays decreased. As the masks were formed by using the solution with a concentration of 10 wt % PS nanospheres, as Figure 8d shows, only small Au-Ag nanopariticle arrays with ordered arrangements were observed. These results prove that 8 wt % PS nanospheres is the optimum concentration for the solution to form the PS nanosphere masks by the drop-coating method.

### 3.3. Selection of the Physical Vapor Deposition Method

**Figure 9:**
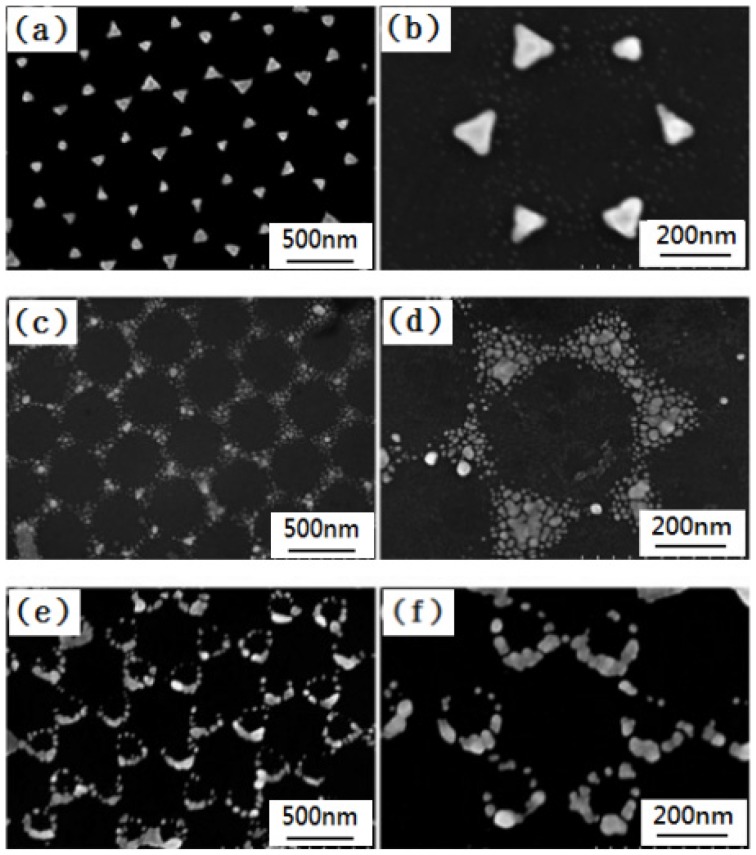
SEM images of two-dimensional ordered Au-Ag nanoparticles arrays fabricated by different physical vapor deposition methods. (**a**,**b**) Thermal evaporation; (**c**,**d**) RF magnetron sputtering; (**e**,**f**) e-gun deposition.

Next, the PS nanosphere masks were fabricated by the drop-coating method by using solution with a concentration of 8 wt % PS nanospheres. Figure 9 is the SEM of two-dimensional ordered hybrid Au-Ag nanoparticle arrays, fabricated by three different physical vapor deposition methods. Figure 9a,b are the SEM images of hybrid nanoparticle arrays deposited by thermal evaporation, with different magnifications. Figure 9c,d are the SEM images of hybrid nanoparticle arrays deposited by RF magnetron sputteringm with different magnifications. Figure 9e,f are the SEM images of hybrid nanoparticle arrays fabricated by e-gun deposition, with different magnifications. From Figure 9, we can view that all of the hybrid Au-Ag nanaoparticle arrays fabricated by the three different deposition methods exhibited an ordered hexagonal distribution, but the structures of each nanoparticle are very different.

**Figure 10:**
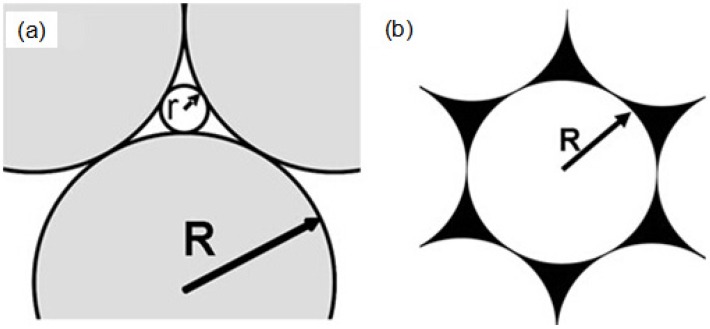
(**a**) Hexagonal close-packed PS nanosphere structure; (**b**) Au-Ag triangle hexagonal nanoparticle arrays after the PS nanosphere masks were removed.

The nanoparticle arrays fabricated by thermal evaporation showed a hexagonal periodic arrangement, which consisted of sharp triangular nanoparticles, and the surfaces of the nanoparticles are very smooth, as Figure 9a,b show. However, the nanoparticle arrays fabricated by RF magnetron sputtering present a triangular honeycomb-like nano-lattice structure and the surfaces of the nanoparticles are unevenly dispersed, as Figure 9c,d show. For sputtering, the vapors of the Au-Ag metals move upward at a broad solid angle. Therefore, the nanotriangles are not connected, as can be observed in Figure 9c,d, although the nanotriangles consist of many smaller nanoparticles, which is perhaps due to the fact that the thicknesses of the deposited Au-Ag films are very thin. The nanoparticle arrays fabricated by e-gun deposition exhibit a triangular ring-shaped nano-lattice structure and most of the metal nanoparticles exist in the triangle edge of the particle surface, as Figure 9e,f show. For e-gun deposition, the vapors of the Au-Ag metals move strictly upwards, showing better directionality. Considering this, the nanoparticle arrays deposited by e-gun deposition will reveal better triangular ring-shaped nano-lattice structures because e-beam deposition is able to produce better-defined nanotriangles [28]. Therefore, in this study, as Figure 9e,f show, the Au-Ag nanoparticles arrays investigated cannot reveal highly ordered Au-Ag triangle hexagonal nanoparticle arrays. In order to explain the results shown in Figure 9, we use a characteristic length to represent the 2D ordered nanoparticle arrays and to describe the theorem fabricated by the three different physical vapor deposition methods. The differences among the Au-Ag nanoparticle arrays fabricated by different physical vapor deposition methods can be analyzed and explained in the following statements. These deposited nanoparticle arrays are carefully observed, and Figure 10a,b are the corresponding structure diagrams. The radius R of the used PS nanospheres is about 265 nm. Using the mathematical calculation, we can determine the relationship between R and r:

(1) $$r=\left(\frac{2\sqrt{3}}{3}-1\right)$$

where R is the radius of the PS nanospheres and r is the radius of the tangent circle in the gap between the PS nanospheres. According to equation (1), the corresponding r is calculated and has a value of about 41 nm. It can be concluded that if the particle’s radius is less than 41 nm, the particle can theoretically pass through the gap between the PS nanospheres. Then, the metal nanoparticles will coat the surfaces of the substrates and combine with the surfaces to form the triangle hexagonal nanoparticle arrays. 

**Figure 11:**
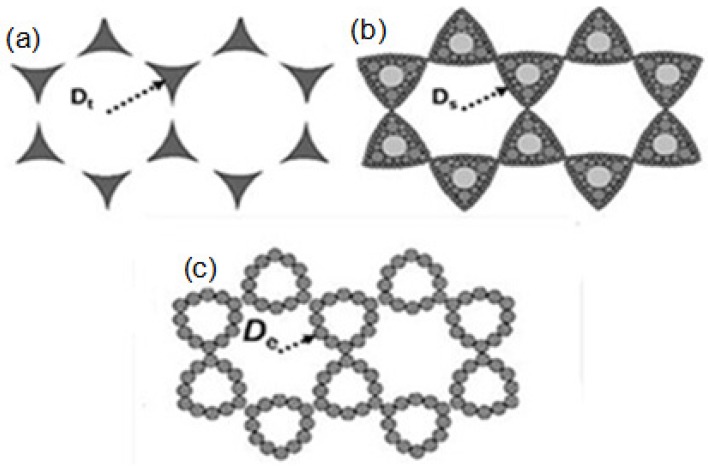
Schematic diagrams of silver nano-lattice structures (**a**) thermal evaporation; (**b**) RF magnetron sputtering; and (**c**) e-gun deposition.

Figure 11 shows three different nano-lattice structures fabricated by three different physical vapor deposition methods and are obtained by the SEM images shown in Figure 9 [29], and the characteristic lengths (*D_t_*, *D_s_*, and *D_e_*) represent the distance between the center of a single hexagonal lattice and the surrounding area of the noble metal; where *D_t_*, *D_s_*, and *D_e_* represent the characteristic lengths of the nanoparticle arrays fabricated by thermal evaporation, RF magnetron sputtering, and e-gun deposition, respectively. According to the electron microscopic scale obtained from Figure 9, the *D_t_*, *D_s_*, and *D_e_* values are about 250, 240, and 200 nm, respectively. When we use different physical vapor methods to deposit the hybrid Au-Ag nanoparticle arrays, the characteristic lengths of the deposited nanoparticle arrays are different. For example, the size of the particles, the heat of vaporization, the density of the particle, and the deposition rate are inconsistent. This is one of the reasons why the nanoparticle arrays have different structures.

It can be seen from Figure 10 that the radius of gaps between the PS nanospheres is 41 nm, which is much larger than the sizes of the nanoparticles shown in Figure 9 and Table 1. Table 1 compares the characteristic parameters of the deposited Au-Ag nanoparticle arrays deposited by the three different deposition methods. Therefore, the nanoparticles generated by the three methods can pass through the gaps into the substrates and combine with them. As we know, during the deposition process the evaporated Au and Ag particles have a higher temperature and a positive curvature, which can be called “hot nanospheres”. However, the gaps between the PS nanospheres and the substrates have a low temperature and negative curvature, which can be thought as “cold nanotraps”. When the “hot nanospheres” and “cold nanotraps” are combined, the difference between the temperature and curvature causes both attraction and penetration. Under the promotion of these effects, the Au and Ag nanoparticles move into the triangular gaps between the PS nanospheres, and they are tightly bonded to the substrates. The deposited nanoparticles form a triangular pyramid structure and thereby form the hybrid Au-Ag nanoparticle arrays.

In the experiment of thermal evaporation, 3.35 eV was used to evaporate the Au and Ag metal nanoparticles, and the surface energy and surface temperature were not too high. However, due to the aggregation size of the metal atoms being less than 10 nm and the deposition rate of about 2.5 nm/s, the density of the deposited particles is high. When a large number of metal nanoparticles arrive at the same time, in a relatively short period of time, the initial temperature is not too high and the nanoparticles are maintained at a very stable state. During the deposition process of thermal evaporation, the nanoparticle monomers will collide with each other and bond together. On the substrates’ surfaces, the metal nanoparticle monomers will not reach the gap depths of the PS nanosphere masks and will be deposited on the vacancies of the formed masks. Considering this, the movement distance of the deposited metal nanoparticles is relatively short. For example, the characteristic length of the structure is 250 nm, which is similar to the value of the radius when using PS nanospheres (265 nm). Therefore, the metal nanoparticles are promoted to obtain more complete triangle hexagonal nanoparticle arrays, as shown in Figure 9a,b.

In the experiment of RF magnetron sputtering, the energy used to heat the metal nanoparticles for evaporation has a high value, of 15 eV. The surface energy and surface temperature are relatively high, and the size of the metal nanoparticles is smaller than that when using the method of thermal evaporation. The density of the metal nanoparticles is relatively large and the nanoparticles are very unstable; they easily collide with other particles and will adhere together. In this process, the metal nanoparticles have the aggregation size of metal atoms of about 10 nm, which is relatively large and cannot move to a distant location. The metal nanoparticles combine with other particles to fix in the middle location of the gap of PS nanosphere masks. In opposition, if the size of the metal nanoparticles is relatively small, such as the particle with a size of about 5 nm, they will have a larger curvature effect. Those metal nanoparticles are particularly active after reaching the substrates, and they can move to a distant location to reach the depths of the gaps of the PS nanosphere masks, and they are finally fixed in a far away location. Thus, the nanoparticle arrays obtained by RF magnetron sputtering exhibit a triangular honeycomb nanostructure with a smaller aggregation size of metal atoms in the outer edges. Considering this, the metal nanoparticles show a roughly dispersed state, as shown in Figure 9c,d. When compared with the thermal evaporation method, the moving distance of the metal nanoparticles in RF magnetron sputtering is relatively large, but it is still limited. The deposition structure with a characteristic length of 240 nm has proven this description.

As the e-gun deposition is used to deposit the Au and Ag thin films, the evaporation heat of the metal nanoparticles is about 4.46 eV, which is relatively low. Meanwhile, **t**he surface energy and the surface temperature are also relatively low. In addition, the deposition rate is slow, at only 0.27 nm/s, and the size of the metal nanoparticles has a relatively large value of about 20–25 nm, for which the density of the metal nanoparticles will have a relatively small value. Considering this, a lower number of metal nanoparticles can reach the substrates to form the nanoparticle arrays. For the deposited metal thin films with a thickness of ~ tens nanometer, the total number of deposited particles is relatively low when using the e-gun deposition. At the same time, the two factors of a lower particle number and lower surface temperature mean that the metal nanoparticles do not easily collide with each other and bond together. The Au and Ag metal nanoparticles have an adequate amount of time to move to the deep location of the gaps between the PS nanosphere masks. When the metal nanoparticles are in the middle of the gap, they will eventually move to the edge of the area, and the center area of the gaps will only contain a few nanoparticles, as shown in Figure 9e,f. For this reason, the nanoparticle arrays fabricated by e-gun deposition exhibit a triangular ring-shaped nano-lattice structure. Based on the above analyses and the considerations of the experimental property and efficiency, we know that some research has used different physical methods (radio-frequency magnetron sputtering or e-gun deposition) to deposit high quality single-layer and multi-layer periodic metal nanoparticle arrays by using the NSL method. Therefore, in this study, we find that thermal evaporation can also be used to deposit the Au-Ag nanoparticle arrays with a high uniformity. The results suggest that if we control the parameters to form the PS nanosphere masks and consider the experimental cost and property, we can deposit high quality Au-Ag nanoparticle arrays by using the low coat method of thermal evaporation.

## 4. Conclusions

In this study, the parameters used by the polystyrene (PS) nanospheres to form the masks of the nanosphere lithography (NSL) method were well investigated. At first, we found that the drop-coating method was better than the spin-coating method, because the matrixes of the PS nanosphere masks fabricated by using the drop-coating method revealed a more uniform matrix and smaller gaps. A value of 10° was the optimum one-side lifting angle, producing self-assembly nano-scale PS nanosphere masks with a high uniformity and densification. We also found that 40 and 55 °C were the best temperatures to employ for the vaporization of solvent in the nano-scale solution of PS nanospheres. A value of 8 wt % was the optimum concentration of PS nanospheres for the solution of the drop-coating method, because the PS nanosphere masks revealed a hexagonal close-packed structure and almost no dislocations or vacancies. As RF magnetron sputtering was used to deposit the Au and Ag thin films, the deposited nanoparticles formed a triangular honeycomb nanostructure with smaller particles being roughly dispersed in the outer edges. Moreover, as the e-gun deposition was used to deposit the Au and Ag thin films, the deposited nanoparticles formed a triangular ring-shaped nano-lattice structure. Finally, as thermal evaporation was used to deposit the Au and Ag thin films, the deposited nanoparticles formed a triangular structure and thereby formed uniformly hybrid Au-Ag nanoparticle arrays. The results in this study prove that if the PS nanospheres are well deposited as a highly uniform NSL mask, thermal evaporation can be the best method to deposit the hybrid Au-Ag nanoparticle arrays, rather than RF magnetron sputtering and e-gun deposition. 

## Figures and Tables

**Table 1:** Comparison of characteristic parameters of the deposited Au-Ag nanopariticle arrays by the methods of thermal evaporation, RF magnetron sputtering, and e-gun deposition.

Coating Technology	Thermal Evaporation	RF magnetron Sputtering	E-gun
Aggregation size of metal atoms	10 nm	5–10 nm	20–25 nm
Evaporation heat	3.35 eV	15 eV	4.46 eV
Particle density	large	Relatively large	small
Deposition rate	2.5 nm/s	0.11 nm/s	0.27 nm/s
